# FEASIBILITY AND ACCEPTABILITY OF A VIRTUAL EXERCISE PROGRAM IN OLDER ADULTS WITH PREDIABETES

**DOI:** 10.1093/geroni/igae098.1030

**Published:** 2024-12-31

**Authors:** Kathryn Porter Starr, Elizabeth Kemp, Eric Levitan, Nathan Boucher

**Affiliations:** Duke University School of Medicine, Hillsborough, North Carolina, United States; Vivo, Atlanta, Georgia, United States; Vivo, Atlanta, Georgia, United States; Duke University, Durham, North Carolina, United States

## Abstract

Through metabolic and physiological changes, aging and insulin resistance precipitate impaired physical function and its progression to type 2 diabetes (T2DM). Further, individuals with T2DM have increased risk for functional disability and mobility limitation. Therefore, the purpose of this study was to evaluate the feasibility and acceptability of Vivo, a commercially available virtual exercise program, for older adults at risk of developing T2DM. A priori criteria for feasibility and acceptability were defined as ≥80% of participants would be retained, ≥80% would attend all 24 classes and ≥90% of the program would be delivered as designed. In this 12-week single arm feasibility study, 28 older adults (69±6 years; 58% female; and 73% non-Hispanic white) with prediabetes were recruited. Out of the 28 individuals enrolled, 22 (79%) successfully completed the 12-week intervention. The results revealed a 27% increase in upper body strength (30-sec arm curl), a 13.5% increase in lower body strength (30-sec chair stand), and a 33% improvement in endurance (2-minute knee raise). Furthermore, the mean change in the 30-second chair stands was 2.0±2.2, with 14 participants increasing by ≥2 stands. Of the 22 participants who completed the intervention, 85% attended all 24 classes. Finally, intervention fidelity was assessed using a 9-item checklist with four focus areas; intervention delivery, workout intensity, safety, and participation and engagement. The intervention was delivered with 96% fidelity across three Vivo trainers. Study findings illustrate that online, live virtual exercise programs that provide tailored programming are highly feasible and acceptable and result in meaningful functional improvements.

